# *Notes from the Field: Escherichia coli* O157:H7 Outbreak in Children with *Clostridioides difficile* Colonization Associated with an Improperly Treated Swimming Pool — Pennsylvania, June 2021

**DOI:** 10.15585/mmwr.mm7120a4

**Published:** 2022-05-20

**Authors:** Molly E. Nace, Jennifer L. Wallace, Kelly E. Kline, Nottasorn Plipat

**Affiliations:** 1 Bureau of Epidemiology, Pennsylvania Department of Health.

On June 7, 2021, the Pennsylvania Department of Health (PADOH) received multiple complaints of gastrointestinal illness from patrons of a community swimming pool. Two patrons reported positive Shiga toxin-producing *Escherichia coli* (STEC) and *Clostridioides difficile* from stool specimens. PADOH issued pool closure orders and initiated an outbreak response to identify a source and prevent additional illnesses.

Confirmed cases were defined as isolation of *E. coli* O157:H7 or detection of Shiga toxin or Shiga toxin genes from stool specimens of persons who visited the pool during May 31–June 7, 2021. Probable cases were defined as three or more loose stools in 24 hours with nausea, vomiting, fever, or cramps in persons who visited the pool during the same time frame. *C. difficile* results were deemed incidental upon consultation with experts (LC McDonald, MD, CDC, personal communication, June 2021) and were not included in the case definition.

Fifteen cases (nine confirmed, six probable) in persons aged 4–14 years were identified; 10 patients were male (Table). All persons reported swimming at the pool on May 31, 2021, the seasonal opening date, and had no other common exposures. The total number of pool visitors on this date is unknown. Symptom onsets occurred during June 2–June 4, 2021. Thirteen patients sought medical evaluation, and six were hospitalized. Four received antibiotics for *C. difficile*. None developed hemolytic uremic syndrome.

**TABLE:** Laboratory and clinical details for patients associated with an *Escherichia coli* O157:H7 outbreak (N = 15), including three presumed to be colonized with *Clostridioides difficile* — Pennsylvania, June 2021

Patient	*E. coli* O157:H7 result			*C. difficile* result*			Treatment	Hospitalized
	Culture	Shiga toxin (EIA)	Shiga toxin (PCR)	GDH	Toxin A/B (EIA)	Toxin DNA (PCR)		
A^†^	Pos	Pos	NT	Pos	Neg	Pos	Vancomycin	No
B^†^	NT	NT	Pos	NT	Neg	Pos	Azithromycin	Yes
C	NT	NT	NT	Neg	Neg	NT	None	No
D^§^	Pos	Pos	NT	NT	NT	NT	None	No
E^§^	NT	NT	NT	NT	NT	NT	None	No
F	Pos	Pos	NT	NT	NT	NT	None	No
G	Pos	Pos	NT	Neg	Neg	NT	None	Yes
H	NT	NT	NT	NT	NT	NT	None	No
I^†,§^	NT	NT	NT	Pos	Pos	NT	Metronidazole	No
J^§^	NT	NT	NT	NT	NT	NT	Metronidazole	No
K^§^	Pos	Pos	NT	NT	NT	NT	None	Yes
L^§^	Neg	NT	NT	NT	NT	NT	None	Yes
M	Pos	Pos	NT	Neg	Neg	NT	None	Yes
N^§^	Pos	Pos	NT	Neg	Neg	NT	None	No
O^§^	Pos	Pos	NT	NT	NT	NT	Cefoxitin^¶^	Yes

**Abbreviations:** EIA = enzyme immunoassay; GDH = glutamate dehydrogenase; Neg = negative; NT = not tested; PCR = polymerase chain reaction; Pos = positive.

* Type of testing performed varied by laboratory. The laboratories testing patients A, C, G, I, M, and N performed *C. difficile* GDH and toxin EIA, with reflex to PCR when GDH and toxin results were discordant. The laboratory testing Patient B, who had the first reported case, performed *C. difficile* toxin DNA PCR testing first. When the test resulted positive, toxin EIA was then performed. ^†^ Patient was presumed to be colonized with *C. difficile.*  ^§^ Patients D and E, I and J, K and L, and N and O are sibling pairs. ^¶^ Patient received a diagnosis of appendicitis and received 1 dose of preoperative cefoxitin before the appendectomy.

Early findings suggested an unusual association between exposure to a chlorinated swimming pool and infections caused by two pathogens susceptible to chlorine. Pool inspection revealed an automatic chlorinator malfunction. Record-keeping was inconsistent with local requirements, and the few available records demonstrated at least one instance of no detectable chlorine. The pool reopened following chlorinator repair, after which no additional cases were identified.

The investigation highlighted three important points regarding evaluation of outbreaks of childhood diarrheal disease. First, *C. difficile* testing is only recommended for children aged ≥2 years with prolonged or worsening diarrhea and risk factors, including immunocompromising conditions or relevant exposures (e.g., recent health care visits or antibiotics).* Reported prevalence of asymptomatic *C. difficile* colonization might vary by study population, laboratory detection method, and environmental setting. One study of children aged 1 month–12 years with diarrhea identified *C. difficile* toxin B in 3% of outpatients, 5% of inpatients, and 7% of asymptomatic controls (*1*). Recent studies using molecular techniques reported rates up to 25% in asymptomatic children aged 1–5 years (*2*) and 24% in persons aged 1–18 years without diarrhea (*3*). In the current outbreak, all children were previously healthy and considered to be at low risk for *C. difficile* infection. Thus, *C. difficile* testing was not indicated and provided no relevant clinical or epidemiologic data. Second, laboratory reports should include age-based interpretive suggestions for colonization versus infection and reminders that clinical symptoms are required for a diagnosis of *C. difficile* infection. Provider interpretations should include clinical and epidemiologic information. Finally, antibiotics are usually not required for treatment of diarrheal illnesses. In this STEC outbreak, no adverse outcomes were reported among the children receiving antibiotics. However, among STEC-infected persons, current guidance recommends against antibiotic use because of the risk for hemolytic uremic syndrome (*4*). 

Enteric disease outbreaks caused by multiple pathogens rarely occur. Coinfections with *C. difficile* and other pathogens are unusual, but possible (*5*). Full investigation revealed that this outbreak was likely the result of STEC infections among children, some of whom were colonized with *C. difficile*. Recreational waters should be properly treated and maintained,^†^ and persons experiencing diarrhea should abstain from swimming.

## Footnotes

*
https://academic.oup.com/cid/article/66/7/e1/4855916
